# The impact of a combinatorial digital and organisational intervention on the management of long-term conditions in UK primary care: a non-randomised evaluation

**DOI:** 10.1186/s12913-019-3984-6

**Published:** 2019-03-12

**Authors:** David G. Lugo-Palacios, Jonathan Hammond, Thomas Allen, Sarah Darley, Ruth McDonald, Thomas Blakeman, Peter Bower

**Affiliations:** 1 Manchester Centre for Health Economics, University of Manchester, Jean McFarlane Building, Oxford Road, Manchester, M13 9PL UK; 2 Centre for Primary Care and Health Services Research, University of Manchester, Williamson Building, Oxford Road, Manchester, M13 9PL UK; 3 Centre for Primary Care and Health Services Research and Alliance Manchester Business School, University of Manchester, Williamson Building, Oxford Road, Manchester, M13 9PL UK; 4 NIHR Collaboration for Leadership in Applied Health Research and Care, Centre for Primary Care and Health Services Research, University of Manchester, Manchester, M13 9PL UK; 5 NIHR School for Primary Care Research, Centre for Primary Care and Health Services Research, Division of Population Health, Health Services Research and Primary Care, School of Health Sciences, Faculty of Biology, Medicine and Health, University of Manchester, Manchester Academic Health Science Centre, Williamson Building, Oxford Road, Manchester, M13 9PL UK

**Keywords:** Combinatorial intervention, Management of long-term conditions, Primary care, NHS test beds

## Abstract

**Background:**

Better management of long-term conditions remains a policy priority, with a focus on improving outcomes and reducing use of expensive hospital services. A number of interventions have been tested, but many have failed to show benefit in rigorous comparative research. In 2016, the NHS Test Beds scheme was launched to implement and test interventions combining digital technologies and pathway redesign in routine health care settings, with each intervention comprising multiple innovations to better realise benefit from their ‘combinatorial’ effect. We present the evaluation of one of the NHS Test Beds, which combined risk stratification algorithms, practice-based quality improvement and health monitoring and coaching to improve management of long-term conditions in a single health economy in the north-west of England.

**Methods:**

The NHS Test Bed was implemented in one clinical commissioning group in the north-west of England (patient population 235,800 served by 36 general practices). Routine administrative data on hospital use (the primary outcome) and a selection of secondary outcomes (data from both hospital and primary care) were collected in the intervention site, and from a comparator area in the same region. We used difference-in-differences analysis to compare outcomes in the NHS Test Bed area and the comparator after initiation of the combinatorial intervention.

**Results:**

Tests confirmed the existence of parallel trends in the intervention and comparator sites for hospital outcomes for the period April 2016 to March 2017, and for some of the planned primary care outcomes. Based on 10 months of post-intervention secondary care data and 13 months post-intervention primary care data, we found no significant impact on primary outcomes between the intervention and comparator site, and a significant impact on only one secondary outcome.

**Conclusion:**

A combinatorial digital and organisational intervention to improve the management of long-term conditions was implemented across a whole health economy, but we found no evidence of a positive impact on health care utilisation outcomes in hospital and primary care.

**Electronic supplementary material:**

The online version of this article (10.1186/s12913-019-3984-6) contains supplementary material, which is available to authorized users.

## Background

Long-term conditions represent a major burden on patients and the health care system. They are estimated to account for 70% of the NHS budget [1] and have a significant impact on health-related quality of life [2, 3].

Current provision of care for people living with long-term conditions has a number of limitations. Services may be designed to respond to individual patient need and to react to crises, rather than planning and organising care for a population over the longer term [4]. Care may not be delivered in accordance with evidence based practice [5], and patients may not experience choice and shared decision-making [6, 7]. Early intervention and prevention of long-term conditions may not receive sufficient attention [8].

Better management of long-term conditions remains a policy priority. In the UK NHS, a wide variety of interventions and policies have been undertaken to improve prevention and management of long-term conditions. Much attention has been paid to improvements in self-management, such as programmes like the Expert Patients Programme [9]. Service-level interventions include care planning [10] and case management [11], as well as the introduction of pay for performance [12]. There has also been significant attention to the potential for digital technology to augment impact, through the adoption of risk stratification models and assistive technology [13]. Although some of these have been associated with improved outcomes, few have demonstrated benefits consistently so as to support widespread adoption, especially in terms of the outcome of reduced hospital use [14–16].

A number of factors may have been responsible for the lack of effects from previous interventions, including suboptimal implementation, lack of sensitivity to the particular context into which health services interventions are implemented [17, 18], and the difficulties of changing outcomes in patients who often have significant needs [8] . Another criticism is that interventions have often been introduced in relative isolation, as part of a formal experiment. Although this provides maximum levels of methodological control, it fails to take advantage of synergies between different interventions, and their ability to affect change at multiple levels within health care (patients, professionals and the health care system) to maximise effects [19].

The NHS Test Beds scheme was launched by NHS England in 2016 [20]. Wave 1 of the scheme was designed to support the introduction and implementation of interventions involving digital technologies and pathway redesign at scale within routine NHS services by partnerships of NHS organisations and innovators. The objective is to take advantage of the ‘combinatorial’ nature of the impacts of the individual innovations to achieve changes of greater magnitude than have been found in previous evaluations of individual programmes.

### A combinatorial intervention for long-term conditions

Our aim was to assess the impact of a primary care-based NHS Test Bed, which was focussed on the management of three long-term conditions: chronic obstructive pulmonary disease (COPD), type 2 diabetes and heart failure. This combinatorial intervention sought to achieve benefit through three components. The first was risk stratification, using routine NHS data to identify groups of patients on the basis of their predicted risk of poor outcomes. Although risk stratification is a common tool in population-based care for long-term conditions, the evidence for its effectiveness is limited, either as a stand-alone intervention [15] or when linked to wider case-management or integrated care initiatives [11, 16]. The second component was quality improvement involving audit and feedback. Audit and feedback is defined as ‘summary of the clinical performance of healthcare provider(s) over a specified period of time’ [21] and is often combined with other interventions (such as professional education) to identify gaps in care and encourage changes to care to overcome these identified gaps. There is extensive evidence for the effectiveness of a range of quality improvement initiatives which include audit and feedback [21–23], although the impact of individual interventions is often modest. The third component was telemonitoring and health coaching. Health coaching is defined as an intervention which ‘aims to enhance patients’ self-management abilities by providing information for a better understanding of their conditions, to improve the ability to collaborate with health care providers, and to use goal setting related to disease management’ [24]. It is sometimes combined with telemonitoring, which provides technology to allow patients and professionals to share information for monitoring and assessment of outcomes. There is some evidence for the effectiveness of telehealth in these conditions [25, 26] although it has proved more difficult to achieve these benefits at scale [27, 28]. Similarly, the evidence for the effectiveness of health coaching is mixed [24, 29, 30].

It was hypothesised that the combinatorial intervention, delivered over a 12-month period in a single health economy, would deliver greater impact on outcomes than the individual components alone. The proposed ‘combinatorial’ effects of these interventions may occur through a number of mechanisms. Implementation of three interventions across an entire health economy during a relatively short period of time may show combinatorial effects which reflect the simple *additive* benefits of the individual interventions. Combinatorial effects may also be *interactive*. For example, quality improvement efforts may be delivered more effectively through better risk stratification of the patient population, or patients identified through risk stratification might then be referred to health coaching. Specifically, this NHS Test Bed aimed to reduce length of hospital stay, the number of emergency admissions, the number of attendances at accidents and emergencies (A&E) services, the number of outpatient visits and the costs from prescriptions.

Our aim was to assess the impact of this NHS Test Bed on health services utilisation using routine data and a non-randomised comparator.

## Methods

We report the study in line with the SQUIRE guidelines for reports of system level interventions to improve health care [31].

### The local context

The study was conducted in Heywood, Middleton and Rochdale Clinical Commissioning Group (HMR CCG). CCGs are statutory NHS bodies led by clinical staff bearing responsibility for planning services for their local patient population. The area covered by HMR CCG is one of the most deprived in England. The population has a high prevalence of long-term conditions (such as COPD, diabetes and heart failure) associated with significant impacts on health and health care costs.

### Ethics

The current study represents a service evaluation. There was no randomisation or delivery of treatment by protocol, and most of the individual services under test had all been commissioned for use in the NHS previously, and were being delivered as part of commissioned local service delivery. Therefore, formal ethical approval was not sought for the aspects of the evaluation reported here using routine data, although other aspects (such as interviews with professionals for a process evaluation, reported elsewhere) did receive ethical approval (Reference 2017–2150-3284).

### Intervention - the NHS test bed

The HMR CCG Long Term Conditions Test Bed (hereafter ‘Test Bed’) aimed to improve care of patients with COPD, diabetes and heart failure. The Test Bed had three core components.

(i) *Clinical audit and population management software including risk algorithm*

Clinical audit and population management software (*MSDi Optimise*) for primary care professionals provided modules for general records management to support the on-going organisation of care, data on patient risk of emergency hospital admissions (*QAdmissions*), and an algorithm that provided prediction scores about patients’ risk of developing long-term conditions (COPD, heart failure, type 2 diabetes) at 12 and 24 months.

(ii) *Quality improvement*

A structured 12-month ‘clinical change management programme’ (*Evidence into Practice*) for COPD and type 2 diabetes was used to inform and upskill primary care teams. The intervention involved three steps (a) understanding current practice through the review of ‘Care In Practice’ reports (one at the start of the programme, at 6 and 12 months), and ‘confidence mapping’ questionnaires, based on audits of GP practices’ own data and highlighting areas where there was scope for patient care to be improved (b) review and implementation of guidelines through a clinical change management process supported by facilitators (c) measurement of progress.

(iii) *Telehealth monitoring and coaching*

A remote telehealth monitoring and coaching service was provided for patients with heart failure or COPD (*Closercare*). Heart failure patients were identified from the caseloads of local heart failure nurses and enrolled primarily by them, whereas COPD patients were predominantly identified through the running of searches on GP practice EMIS systems and enrolled within primary care. The telehealth intervention involved initial remote monitoring for patients (blood pressure, blood oxygen, weight and self-report questionnaires) using a smart phone and a variety of other equipment for 12 weeks where patients submitted their readings every morning, followed by 6 weeks of telephone health coaching (one 60 min session per week) based on the ‘activation’ model [32].

### The test bed partners

The Test Bed team comprised HMR CCG, MSD, and Verily. HMR CCG was the lead organisation and employed the programme director. Each organisation hosted a programme team, and these teams were in frequent communication and took part in regularly scheduled meetings. Three ‘Change Managers’ (one employed by the CCG, and two by MSD) operated within HMR for the majority of the Test Bed, each working with 10 GP practices. They had a crucial role ‘on the ground’ in primary care, engaging practices and supporting them with activities related to all three Test Bed components. Additional organisations – BARDOC, Healthcare at Home, and Pennine Acute Trust – were contracted to facilitate and deliver different aspects of Closercare.

### Evaluation approach and statistical analysis

We evaluated the effects of the intervention using a non-experimental approach – (*difference in differences* – DiD) [33], with the planned methods outlined in a protocol (see Additional files 1 and 2). This method requires distinct intervention and comparator groups, as well as data on relevant outcome before and after implementation of the intervention. DiD analysis quantifies the change in the outcome in the intervention site against a comparator, to account for changes in outcomes over time which would have been expected if there were no intervention. The key assumption of DiD is that the intervention and comparator site follow the same trend in the outcome variable.

Parallel trends are assessed visually by plotting outcome variables over time for each treatment and comparator regions. Additionally, a statistical test of the existence of parallel trends across regions is conducted. Where the null hypothesis of parallel trends is not rejected, the DiD model is estimated. Specifically, the DiD model regresses an outcome variable on a time effect and a dummy variable indicating the post-intervention period for HMR CCG. The coefficient on this dummy measures the intervention effect.

### Data sources

Data from hospital and primary care sources was provided from HMR CCG, and we negotiated with local CCGs in the same region of England for access to equivalent data. The comparator site able to supply equivalent data was Bury CCG which is also part of the Greater Manchester Health and Social Care Partnership [34]. The table in Additional file 3 compares the health profiles of the population in HMR and Bury CCG catchment areas. HMR is significantly more deprived than Bury, with a higher proportion of ethnic minority patients. Despite these differences, indicators of the health of the population in terms of health behaviours (such as smoking and activity) and disease prevalence (proportions with long term conditions) are very similar, and the practices in the Test Bed sites and the comparator achieve similar levels of quality of care for long-term conditions (as defined by NICE).

Primary care data was extracted from general practices in HMR and Bury CCGs from patient records data using Read Codes to define activity. Read Codes are the standard clinical coding system used to record summary clinical and administrative data for GPs within the NHS [35]. The secondary care data contained patient records of A&E visits, outpatient visits and hospital admissions. The secondary care data was from April 2011 to January 2018 while the primary care data was from April 2011 to April 2018.

We planned to extract data on a range of outcomes which were hypothesised to be amenable to change as a result of the intervention, and where routine data were available. Due to a variety of issues, not all the outcomes planned in the protocol were analysed, and the changes are detailed in Table 1.

**Table 1:** Intended and actual outcomes

	Outcome in the protocol	Outcome analysed
1	Emergency admissions for a Test Bed condition (pre-defined primary outcome)	Analysed in line with protocol
2	Emergency admissions for any condition	Analysed in line with protocol
3	Emergency admissions for ambulatory care sensitive conditions (ACSCs)	Analysed in line with protocol
4	Emergency attendances	Analysed in line with protocol
5	GP referrals for outpatient appointments	Analysed in line with protocol
6	Number of patients achieving NICE clinical standards of care	Not analysed
7	Service utilisation: pulmonary rehabilitation	Inhaler technique observed by GP Use of spirometry by GP
8	Service utilisation: cardiac rehabilitation	Use of echocardiogram by GP
9	Service utilisation for smoking cessation	Smoking cessation advice recorded by GP
10	Service utilisation: diabetes management	Referral by GP to diabetes education programme

Outcomes 1–5 are generated from secondary care data, while outcomes 6 to 10 are generated from primary care data using Read Codes. We hypothesised that indicators 1–5 should decrease due to the intervention while indicators 6–10 should increase. Data sharing was based on a detailed agreement and secure facilities at the University of Manchester to enable safe data storage.

Due to stipulations in data sharing agreements, the patient-level data had to be aggregated to several different levels (practice-month, practice-quarter and CCG-month). Aggregation involved censoring values which were below 10 in any given level. This censoring rule was applied to all aggregations of the data but the CCG-month data was affected least and therefore is used in our analysis. Data aggregation meant that certain outcomes in the protocol could not be analysed (pulmonary rehabilitation, cardiac rehabilitation, diabetes weight management services, and NICE clinical standards). It should be noted that the fact that certain outcomes were censored indicates that they were not frequently used.

## Results

### Description of the implementation of the intervention

There are 36 practices in the CCG, of which 31 signed up to Test Bed. Practices reported usability problems with the initial version of the clinical audit and management software (*MSDi Optimise)*, and a second version was implemented to address these, followed by a third version, which included the long-term condition risk algorithm. Some of the early usability issues meant that there was minimal use of the software to identify prospective patients for Closercare, as intended, and minimal evidence of practices regularly using the software for other purposes for which it was suitable (e.g. to inform the content of team meetings about patients at risk of hospital admissions, or prompt invitations of specific patients to the practice for a medication review). A number of delays to the installation of version 3 on practice systems meant that there was only a limited period (around 2 weeks to 1 month depending on the practice) in April–May 2018 for practices to use the risk algorithm.

The quality improvement (*Evidence into Practice)* component of the intervention was completed by 30 out of 31 participating practices. Each practice participated in three review meetings, at commencement, 6 and 12 months. The Test Bed team reported that a range of practice staff including nurses and GPs attended most of these sessions. As part of the intervention, clinical educational sessions for COPD and diabetes were provided by specialists for all practices. The diabetes sessions involved a local expert visiting each practice over a 12-month period to provide an update on the latest diagnostic and treatment guidelines and offer bespoke advice to nurses and GPs. A COPD expert from outside the area ran evening sessions for all practices in late 2017 and early 2018. This included advice and guidance on proper inhaler technique, appropriate prescription of rescue packs, and providing micro-spirometry. Several practices requested additional COPD training sessions, which were provided by the same expert and tailored to the specific needs of their clinical teams. The most commonly identified benefits of involvement in the component overall for practices included improvements to coding and patient data quality, increased referrals (e.g. to pulmonary rehab), and identifying particular cohorts of patients that would benefit from additional clinical attention.

A total of 592 patients were enrolled in Telehealth Coaching and Monitoring (*Closercare*), out of the original enrolment target of 1000 (it proved impossible to collect data on the numbers who were eligible or invited; however, the actual rate of participants represents about 1 in 15 HMR residents with diagnosed COPD in 2018 as opposed to the original target, roughly 1 in 10 – see demographics data in Additional file 3). Implementation of the Test Bed was delayed by 9 months due to protracted contract and governance negotiations (which resulted in a 3-month extension), and GP practice engagement in enrolling patients was lower than anticipated (although additional resources were allocated to support this part way through), which help to explain this missed target. However, the number of users completing the whole telehealth coaching and monitoring process was far lower (172, 29%). A number of factors likely contributed to this: the monitoring phase was ended prematurely because a supplier of peripherals and data services became insolvent; some patients struggled to use the monitoring equipment; others did not wish to participate in coaching, and some were discharged early for a variety of reasons (e.g. communications challenges due to their level of spoken English).

### Descriptive statistics of HMR and Bury CCGs

Tables 2 and 3 present descriptive statistics for our hospital and primary care outcomes. Each cell contains the total incidence of the outcome occurring in each CCG in a specific period of data collection. HMR CCG serves a larger population. Hospital outcomes are grouped into periods covering April 2016 to March 2017 (the year before the Test Bed), and April 2017 to January 2018 (the 10-month period since the start of the Test Bed). Primary care outcomes cover April 2016 to March 2017 and April 2017 to March 2018. Our analysis accounts for the shorter post-intervention period for the secondary care outcomes.

**Table 2:** Descriptive statistics for secondary care outcomes

	HMR		Bury	
	April 2016–March 2017	April 2017–January 2018	April 2016–March 2017	April 2017–January 2018
Test Bed condition admissions (Primary Outcome)	1160	1048	804	675
All emergency admissions	25,187	22,920	19,369	17,274
Admissions for ACSCs	2228	1999	1678	1453
Emergency attendances	97,818	84,767	62,138	55,798
GP referrals for outpatient appointment	56,790	47,810	44,047	36,152

**Table 3:** Descriptive statistics for primary care outcomes

	HMR		Bury	
	April 2016–March 2017	April 2017–March 2018	April 2016–March 2017	April 2017–March 2018
Smoking cessation advice	36,070	41,771	31,876	27,499
Diabetes education programme	913	848	646	539
Spirometry performed	1610	1784	451	460
Inhaler technique shown	1439	1848	5758	6716
Echocardiogram performed	1115	1315	1856	2208

### Testing for parallel trends in HMR and Bury CCGs

**Fig. 1:**
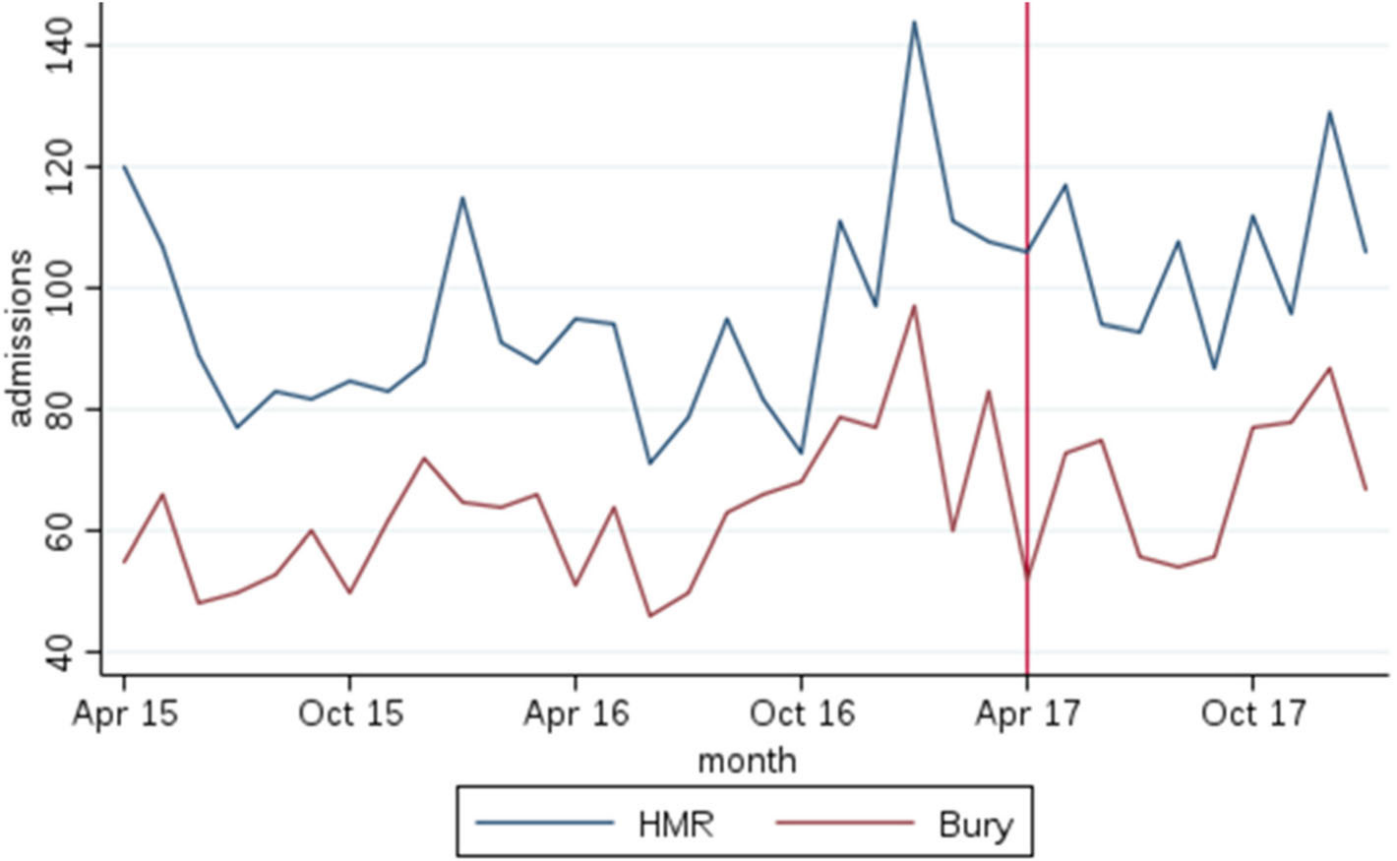
Graphical analysis of parallel trends for emergency admissions for long-term conditions

The DiD method requires testing that outcomes of interest follow parallel trends in the intervention and comparator site. Figure 1 and Additional files 4 and 5 plot the CCG monthly averages for the two sites for each hospital and primary care, with data beginning in April 2015, to allow longer trends to be observed, and ends in January 2018 for secondary care outcomes and April 2018 for primary care outcomes. The start of the Test Bed in April 2017 is marked on the graphs.

From Fig. 1 and Additional file 4, a visual inspection suggests that parallel trends exist for secondary care outcomes before the initiation of the intervention. Statistical tests do not reject the existence of parallel trends between HMR and Bury in all hospital outcomes for the period April 2016 to March 2017. However, over the period, from April 2015, parallel trends are rejected for the emergency attendances outcomes, and thus we present analysis of all outcomes over the same period (April 2016 to January 2018). Analysis was also performed over the longer period for outcomes passing the parallel trends test.

From Additional file 5, a visual inspection is less supportive of parallel trends in primary care outcomes, and only for outcomes relating to smoking cessation advice and diabetes education programmes the statistical tests do not reject the parallel trends hypotheses. DiD models were not estimated for primary care outcomes which did not demonstrate parallel trends.

### DiD analysis

Table 4 shows the results of the DiD models performed on the outcomes passing the parallel trends test. The DiD coefficient indicates the comparative change in the outcome in question, together with 95% CI and *P*-value. The coefficient 7.6 relating to the primary outcome indicates that, in the period April 2017 to January 2018, emergency admissions for a Test Bed condition increased by 7.6 in HMR CCG compared to Bury (95% confidence interval − 3.7 to 19.0). Confidence intervals suggest reasonable precision of the estimated effect in the aggregated CCG-level date. An effect at any point within the 95% confidence interval would not constitute a successful outcome.

**Table 4:** DID results for all six clinical outcomes

	DID	95% CI	*P*-value	Observations	adjusted R^2^
Primary outcome					
Test Bed condition admissions	7.6	− 3.7 to 19.0	0.18	44	0.93
Secondary care outcomes					
All emergency admissions	79.8***	21.2 to 138.4	0.01	44	0.99
Admissions for ACSCs	8.8	−6.1 to 23.6	0.23	44	0.94
Emergency attendances	−76.4	− 264.9 to 112.1	0.41	44	0.99
GP referrals for outpatient appointment	103.9	− 262.3 to 470.1	0.56	44	0.92
Primary care outcomes					
Smoking cessation advice	794.9*	−89.4 to 1679.2	0.08	50	0.85
Referral to diabetes education programme	3.1	−16.6 to 22.7	0.75	50	0.68

* *p* < 0.1, *** *p* < 0.01 Robust standard errors. Only DID coefficients shown, omitted time effects

Of the additional secondary care outcomes in Table 4 only the outcome relating to emergency admissions for all conditions was statistically significant. The results suggest an increase of 79.8 admissions during the intervention (95% CI of 21.2 to 138.4).

In the primary care outcomes, only smoking cessation advice was found to increase between April 2017 and April 2018 in HMR CCG. The estimated increase was 794 (95% CI -89.4 to 1679.2). Further examination of the trends suggests that this observed increase in smoking cessation advice in HMR may have been the result of a sudden and substantial decrease in Bury.

## Discussion

In summary, our evaluation found that the NHS Test Bed has not had the expected effect on either the primary or secondary outcomes. The only statistically significant effect on outcomes showed an increase in all emergency admissions, but it should be noted that the increase is small (estimated at around 80 additional admissions against a total of 25,187 over that period). The finding that interventions of this type increase admissions has been reported previously [8, 11, 15, 36]. It is not clear what causes such increases, although a common hypothesis is that interventions identify additional unmet needs in this population of patients [11].

### Limitations of the evaluation

First, we consider the limitations of the methods adopted in the evaluation. This was a pragmatic study of the effects of a complex, combinatorial intervention on policy relevant outcomes in routine NHS settings. Although the PRECIS-2 tool is designed for trials [37], our evaluation met many of the criteria for a pragmatic study, including inclusion of the whole CCG population for the assessment of outcomes, no strict eligibility criteria, and flexibility around adherence to the intervention. The evaluation thus represents a reasonable assessment of the impact of the intervention in routine NHS settings, albeit with the restrictions inherent in the use of a single health economy.

We presented data comparing the populations of the Test Bed and comparator sites. HMR is significantly more deprived than Bury, with a higher proportion of ethnic minority patients. Despite these differences, indicators of the health of the population in terms of health behaviours (such as smoking and activity) and disease prevalence (proportions with long term conditions) are very similar, and the practices in the Test Bed sites and the comparator achieve similar levels of quality of care for long-term conditions (as defined by NICE). It should be noted that the DiD method is not based primarily on assumptions about the comparability of the underlying patient populations, but the pre-test trends in outcomes. The test conducted for the DiD demonstrated that pre-Test Bed trends in outcomes were also similar, which suggests that the comparator provided a rigorous test of the effects of the intervention.

The main comparison did not have the protection of randomisation. Although a randomised trial would have been possible in principle, it was not practically feasible given funding limitations and the timelines for the Test Bed. DiD is a recognised alternative when randomisation is not possible [33], but we cannot rule out the possibility of unmeasured confounding. The choice of comparator site was largely based on our ability to access equivalent data, although we have presented additional data on site characteristics, and tested for similarity in pre-intervention trends.

There was no formal pre-study specification of sample size, as we were limited to a single CCG and comparator. For this reason, the number of observations is low (44 or 50) as the analysis is performed on data aggregated to CCG-month level. Therefore, attention needs to be given to the precision of the estimates we have presented. In terms of the primary outcome, it seems unlikely that decisions about the impact of the intervention would be different in the range covered by the confidence interval.

We restricted the analysis to routine administrative data, which has significant advantages in terms of data completeness and relevance to the aims of the intervention. However, we were not able to collect data on patient outcomes, and our analysis of impact is therefore a partial assessment of the impact of the Test Bed.

Next, we consider the limitations associated with the intervention. As with any pragmatic study, implementation of the components was not optimal, with lower than planned uptake of the health monitoring and coaching, and limited evidence of engagement with the stratification tools or the clinical and population management software hosting them. This would naturally reduce the effects of any intervention, although some of the issues that occurred may arise in the implementation of comparable technologies in primary care settings where capacity for additional workload is limited.

The HMR Test Bed targeted three prevalent long-term conditions associated with significant burden and health care utilisation. Some patients with one of those conditions would have had at least one of the other two, and many patients would have had additional co-morbidities beyond the three index conditions. However, the intervention did not specifically target patients with multimorbidity, and the Test Bed intervention was not designed to improve the management of multimorbidity per se. It is possible that a more explicit focus on multimorbidity would have led to different results, although the evidence base for interventions in this area is weak [38, 39].

We estimate that the analysis represents approximately 10–12 months of operation of all components of the Test Bed (and hence any ‘combinatorial’ effect), although the limited operational period and use of the long term condition risk algorithm is an important caveat to this. A longer period of use of this algorithm and a longer duration of assessment may provide different results, as the effects of the Test Bed may only become apparent once changes and new clinical routines have been embedded in practice. However, that would have necessarily increased costs of implementation.

### Meaning of the results for policy and practice

We conclude that the Test Bed did implement a complex, multi-faceted intervention across an entire CCG, with reasonably high levels of adoption of the quality improvement component (Evidence into Practice), but more limited adoption of the other two. The quantitative analysis shows that the Test Bed has not had the expected effect on either the primary or secondary outcomes.

Underlying the Test Bed was the suggestion that the simultaneous implementation of multiple innovations would be more likely to lead to improvements in outcomes. This is a reasonable hypothesis, but the evidence from this study suggests that this was not achieved. This may reflect the difficulties of implementing multiple interventions at the same time, where the potential combinatorial power is diluted by the difficulties of implementation among busy professionals’ teams who need to learn new skills and re-design new clinical pathways. Although there were some potential interactive effects between the Test Bed components (such as the clinical audit and management software being used to inform referrals for health coaching), these were not strong features of the Test Bed design or implementation, and the main combinatorial impact anticipated was probably additive. In that case, the additive benefits of multiple components may be lost due to the impact on the ability of teams to implement them in a short period of time, and the specific implementation challenges that occurred in relation to the telehealth and risk stratification elements of the intervention.

The implementation challenges, and adaptations, make drawing conclusions about the feasibility of spreading the Test Bed as a whole over a broader area difficult. Focusing on the components individually, Closercare appears to be the most amenable to spread as logistics, triage, and coaching could be run by a single provider on a broader, perhaps regional, footprint, and this might realise economic savings. However, enrolling a high volume of patients from primary care over a short time necessitated additional resources, which suggests future initiatives might need to relax timescales and/or enrolment targets in the pursuit of cost effectiveness. It is more difficult to see how Evidence into Practice, which relied heavily on the one-to-one relationships between practices and Change Managers could be spread to a larger area with the same resources.

The NHS Long Term Plan states a commitment to expand NHS Test Beds by introducing regional Test Bed Clusters and committing additional resources to evaluating innovations implemented locally within NHS settings [40]. Before this expansion takes place, and when reflecting on the evaluation results of this and other Test Beds, national level orchestrators of the scheme may wish to consider whether the particular emphasis on combinatorial innovation in the design of Test Beds is contributing to implementation challenges in the time and format available.

## Conclusion

A combinatorial digital and organisational intervention to improve the management of long-term conditions was implemented across a whole health economy, with variable uptake of the components across practices and among patients. Although a longer evaluation or more consistent implementation may have demonstrated different results, the analysis presented here found no evidence of a positive impact on health care utilisation outcomes in hospital and primary care.

## Additional files

Additional file 1: Heywood Middleton and Rochdale Long Term Conditions NHS Test-Bed: A service evaluation of implementation and impact. Quantitative study protocol. (DOCX 42 kb)

Additional file 2: Heywood Middleton and Rochdale Long Term Conditions NHS Test-Bed: A service evaluation of implementation and impact. Qualitative study protocol. (DOCX 98 kb)

Additional file 3: Comparing HMR and Bury. Descriptive statistics of HMR and Bury CCGs. (XLSX 11 kb)

Additional file 4: Graphical analysis of parallel trends for secondary care outcomes. Four graphs comparing the trends followed by four secondary care outcomes in both HMR and Bury CCGs. (DOCX 391 kb)

Additional file 5: Graphical analysis of parallel trends for primary care outcomes. Five graphs comparing the trends followed by five primary care outcomes in both HMR and Bury CCGs. (DOCX 566 kb)

## Abbreviations

A&E: Accidents and Emergencies
ACSC: Ambulatory care sensitive condition
CCG: Clinical Commissioning Group
CI: Confidence Interval
COPD: Chronic Obstructive Pulmonary Disease
DiD: Difference in Differences
GP: General Practitioner
HMR: Heywood, Middleton and Rochdale
NHS: National Health Service
NICE: The National Institute for Health and Care Excellence
SQUIRE: Standards for QUality Improvement Reporting Excellence
UK: United Kingdom
