# Supplementary material for: The impact of a combinatorial digital and organisational intervention on the management of long-term conditions in UK primary care: a non-randomised evaluation
Source: BMC Health Serv Res. 2019 Mar 12;19:159. doi: 10.1186/s12913-019-3984-6 (PMC6416963; doi:10.1186/s12913-019-3984-6)
Supplement: Supplementary file 1 — Heywood Middleton and Rochdale Long Term Conditions NHS Test-Bed: A service evaluation of implementation and impact. Quantitative study protocol. (DOCX 42 kb) [file 12913_2019_3984_MOESM1_ESM.docx]

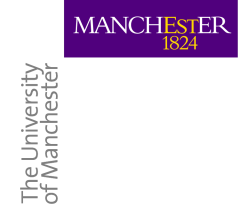


**Heywood Middleton and Rochdale Long Term Conditions NHS Test-Bed**

**A service evaluation of implementation and impact**

| **Protocol version** |  | 1.4 |
| --- | --- | --- |
| **Funding** |  | NHS England via Heywood Middleton and Rochdale CCG |
| **Roles and responsibilities** |  | Principal investigator: Peter Bower  Applicants: Tom Blakeman, Thomas Allen, Matt Sutton, Ruth McDonald  Researchers: Sarah Darley |
|  |  | The funders will have no role in the collection, management, analysis, and interpretation of data; writing of the report; or the decision to submit the report for publication |

**Introduction**

*Background and rationale*

The burden of disease is shifting to long-term conditions, and there is significant interest in the development of interventions that can improve care for these conditions.[^1^](#_1fob9te) Preferably, these interventions need to meet the ‘triple aim’ of improving outcomes, reducing costs, and enhancing patient experience, although at a minimum there is a need to develop interventions that can reduce cost without compromising outcomes and experience.[^2^](#_3znysh7)

A variety of interventions have been tested, including:

- Improvements in the quality of care for long-term conditions through adoption of clinical guidelines and other standard quality improvement methods[^3^](#_2et92p0) [^4^](#_tyjcwt)
- Use of risk stratification models, linked to clinical interventions like case management[^5^](#_3dy6vkm) [^6^](#_1t3h5sf)
- Telehealth, using a variety of models including in-home sensors and more active remote monitoring of long-term conditions[^7-9^](#_4d34og8)

Evidence of effectiveness is variable for all these approaches, and it is unclear what interventions are effective, the populations they are effective in, or the contexts in which they can best achieve change.

There is also increasing consensus that attempting to significantly reduce care costs requires ‘large scale transformations’ rather than the delivery of discrete interventions.[^10^](#_3rdcrjn)

**NHS test beds scheme**

The NHS Five Year Forward View first proposed new NHS Test Beds, which were designed to assess the value of new technologies. The NHS announced the NHS Test Bed Programme in 2016, (<https://www.england.nhs.uk/ourwork/innovation/test-beds/>).

A test bed was described as an area where:

‘Frontline health and care workers in seven areas will pioneer and evaluate the use of novel combinations of interconnected devices such as wearable monitors, data analysis and ways of working which will help patients stay well and monitor their conditions themselves at home’

Heywood, Middleton and Rochdale Clinical Commissioning Group (HMR CCG) successfully led a submission to the NHS England Test-Bed programme looking to pioneer and evaluate the ‘use of novel combinations’ of interconnected innovations to improve patient outcomes at the same or lower cost, when compared to routine care (<https://www.england.nhs.uk/wp-content/uploads/2015/03/test-bed-prospectus.pdf> ).

This Test Bed is in the first wave of programmes announced following a competitive application process and independent external review.

**Heywood, Middleton and Rochdale Clinical Commissioning Group Test Bed**

The Test Bed aims to improve care of patients with COPD, diabetes and heart failure and has 3 core components.

- Clinical audit and population management software for primary care healthcare professionals (*MSDi*).
  - The software provides data on patient risk of emergency hospital admissions, record management and clean-up, and links to other aspects of the Test Bed (referral to Closercare)
- A structured 12 month training programme that upskills primary care teams (*Evidence into Practice*)
  - The intervention involves 3 steps (a) understanding current practice through a ‘Care In Practice’ report (b) review and implementation of guidelines through clinical change management with facilitators , using ‘clinical event review’ and an ‘evidence review’ (c) measurement of progress
- Remote telehealth monitoring service and coaching for up to 1500 patients with Heart Failure or COPD (*Closercare*)
  - This service provides initial remote monitoring for patients (blood pressure, blood oxygen, weight and self-report questionnaire) for 3 months, followed by telephone health coaching based on the ‘activation’ model[^11^](#_26in1rg)

The combination of these interventions, delivered over a 12 month period in a single CCG, are expected to deliver greater impact on health care utilisation (especially hospital admissions) than the individual components alone.

**Evaluation**

Each of the components of the Test Bed has an evidence base from previous evaluations of those components or interventions of the same type, although the scope and quality of the evidence varies.[^5^](#_3dy6vkm) [^12-17^](#_lnxbz9) However, the ‘combinatorial’ effects have not been estimated. The combination of components has the characteristics of a ‘complex intervention’ (as defined by the MRC).[^18^](#_3j2qqm3)

The Test Bed faces two key challenges. Complex interventions are by their nature difficult to implement, as they require changes in service delivery and organisation to be undertaken by professionals and patients in a relatively short period of time.[^19-21^](#_1y810tw) There are many examples in the literature of similar models which have failed to demonstrate effects because of poor implementation or adherence.[^22^](#_1ci93xb)

If the Test Bed achieves reasonable levels of implementation, it remains to demonstrate that those changes in service delivery and organisation will actually lead to significant impacts on outcomes. As noted previously, the evidence that these interventions (alone or combined) translate into robust and enduring changes in health care utilisation is mixed.

**Evaluation objectives**

1. To evaluate the implementation of the Heywood Middleton and Rochdale Long Term Conditions NHS Test-Bed package
2. To assess the impact of the Heywood Middleton and Rochdale Long Term Conditions NHS Test-Bed on hospital admissions and GP referrals

*Study design*

1. Process evaluation using observation and interviews
2. Service evaluation using routine data and a non-randomised comparator

**Methods: Participants, interventions, and outcomes**

*Study setting*

Heywood, Middleton and Rochdale CCG is one of the most deprived areas in England, with high prevalence of the targeted conditions. According to local data, 2% of patients account for 17% of the healthcare spend.

*Eligibility criteria*

The Test Bed is designed to improve the management of patients with COPD, diabetes and heart failure.

As this is a service evaluation rather than a research study, there are no specific inclusion or exclusion criteria beyond those which determine eligibility for the clinical service, alongside patient choice and practitioner clinical discretion.

**Methods**

*Process evaluation of the implementation of the Test Bed*

The MRC defines a process evaluation as:

‘a study which aims to understand the functioning of an intervention, by examining implementation, mechanisms of impact, and contextual factors. Process evaluation

is complementary to, but not a substitute for, high quality outcomes evaluation.’[^23^](#_3whwml4)

Implementation is defined as: ‘the process through which interventions are delivered, and what is delivered in practice. Key dimensions of implementation include: implementation process (the structures, resources and mechanisms through which delivery is achieved); fidelity (the consistency of what is implemented with the planned intervention); adaptations (alterations made to an intervention in order to achieve better contextual fit); dose (how much intervention is delivered); reach (the extent to which a target audience comes into contact with the intervention).[^23^](#_3whwml4) The primary outcome for implementation is how much of the package will be used by clinical staff in routine care in and after the testbed project.

To assess implementation, we will use a combination of observation (of Evidence into Practice training sessions and delivery of CloserCare) and interviews (with primary care staff during and after the roll out of the Test Bed). We will also collect quantitative data on aspects of implementation (start dates, meeting attendance, use of Test Bed components).

We will select six practices in which to conduct qualitative research. This number is a compromise between breadth required to capture sufficient variation and the depth that we need for detailed exploration.

Selection of sites will aim to reflect a range in terms of practice engagement with the programme. We will interview professionals (n=3) at each practice early on in the evaluation and interview these same professionals towards the end of the process (36 interviews in total). We will also observe professionals in each of the sites as they make decisions and take actions in relation to patients who are members of the practice population eligible for the programme.

In addition we will interview staff from MSD and members of the Change Team, as well as CCG representatives. The aim of the interviews and observation is to explore the extent to which the programme is acceptable to health care professionals and the extent to which professionals’ behaviour is in line with that intended by the programme leaders. As part of this process we will identify barriers and facilitators to achieving intended outcomes.

Data collection and analysis will be undertaken concurrently. In addition to notes and reflections on observations combined with interview transcripts, we will use documentary analysis to provide ‘thick description’ of events.[^24^](#_2bn6wsx) Analysis will initially involve coding transcripts using NVivo software and identifying themes. We will use the programme’s intended mechanisms of change to help inform our understanding of the data and focus for data collection. At the same time, we will adopt a sufficiently broad approach to ensure that we do not miss important issues or factors by restricting analysis in too narrow a fashion.

*Service evaluation using routine data and a non-randomised comparator*

The objective of the quantitative evaluation is to assess the impact of the Heywood Middleton and Rochdale Long Term Conditions NHS Test-Bed on hospital admissions and GP referrals. The evaluation will consider the short term impact occurring within the Test-Bed programme.

The implementation of the Test Bed is based on a strict timetable and there is no option for randomisation. A non-randomised design will be used with appropriate comparators.

There is no formal sample size calculation. At present, there are 37 practices and 223,939 patients. Based on Quality Outcomes Framework (QOF) data in 2014/2015, there were 5,276 patients in with COPD, 1,877 with heart failure and 13,741 with diabetes mellitus.

*Outcomes*

Outcomes sourced from linked primary Care-SUS data and aggregated to a monthly series at general practice level.

*Primary Outcome*: Emergency admissions for diabetes, heart failure and COPD.

The Primary Outcome has been selected due to the likelihood that the Long Term Conditions NHS Test-Bed will have greatest impact on emergency admissions for these conditions.

In recognition that the Long Term Conditions NHS Test-Bed will have further impacts beyond the primary outcome the following secondary outcomes will also be analysed.

*Secondary Outcomes:*

1. Emergency admissions
2. Emergency admissions for chronic ambulatory care sensitive conditions
3. Emergency attendances
4. GP referrals for all hospital treatment
5. GP referrals for chronic ambulatory care sensitive conditions
6. GP referrals for diabetes, heart failure and COPD
7. GP level prevalence rates for diabetes, heart failure and COPD
8. Number of patients achieving NICE clinical standards of care
9. Service utilisation for Pulmonary Rehabilitation, Cardiac Rehabilitation Smoking cessation, Diabetes Education/weight management services

*Economic analysis*

Using NHS Reference Costs data, a monetary value will be attached to increases or decreases in hospital activity which are attributed to the Long Term Conditions NHS Test-Bed.

## *Data*

Linked primary Care-SUS data will be provided by MSD under the instruction of Heywood, Middleton and Rochdale CCG and Oldham CCG. Data are at patient level covering all patients registered with HMR or Oldham practices.

Patient level data will be aggregated to practice level to form a longitudinal monthly panel dataset covering the period April 2011 – June 2018.

## *Design*

The intervention will be evaluated using two non-experimental policy evaluation approaches (1) difference in differences (DID) and (2) the lagged dependent variable method. Both methods require distinct treatment and control groups along with distinct pre and post time periods. Figure 1 explains each component for the NHS Test-Bed.

**Figure 1**

|  | **Control** | **Treatment** |
| --- | --- | --- |
| **Pre** | Oldham  2011 – 2016 | Heywood Middleton and Rochdale  2011 - 2016 |
| **Post** | Oldham  2017 – 2019 | Heywood Middleton and Rochdale 2017 - 2019 |

The DID approach does not require that Oldham is a perfect control for Heywood, Middleton and Rochdale, only that they are on the same trends. For example, if once observable differences have been control for (i.e. case mix), Oldham has a higher admission rate when compared to Heywood, Middleton and Rochdale, the DID approach can account for this so long as the trend in admission rate is the same. Changes in outcome between the pre and post periods are calculated for both the control and treatment groups. The difference between these two changes is the effect of the intervention. In our setting, Heywood, Middleton and Rochdale is assumed to follow the same trend as Oldham in absence of the intervention. Any deviation from that trend that we detect can be attributed to the intervention.

If trends in admissions are not similar between Oldham and Heywood, Middleton and Rochdale, the lagged dependent variable method is preferred. This approach differs from DID as it is accounts for differences between the control and treatment group by using information on the outcome in the pre intervention period. In our setting, this information on the pre intervention outcome allows the model to predict what would occur in Oldham and Heywood, Middleton and Rochdale, in the post period, if no intervention was implemented. Any deviation from the predicted trend in the post period for Heywood, Middleton and Rochdale can be attributed to the intervention.

These two approaches can control for hospital and/or practice characteristics that may differ between Heywood, Middleton and Rochdale and Oldham and may also be related to the outcome. Seasonal trends in the outcome can also be accounted for by both approaches.

*Data synthesis*

Where possible we will link the process and outcome evaluations. Data from the process evaluation will be used to assess ‘adherence’ to the intervention in different sites, and may identify the operation of certain contextual mechanisms that moderate the effects of the Test Bed interventions. These may suggest secondary, exploratory analyses.

## Test Bed timescales

- April 2017 – Test Beds begins
- June 2018 – Test Beds ends

**Report timescale**

- September 2017 – National Test Beds Programme Report. Including:
  - Early qualitative analysis in HMR
  - Descriptive analysis of quantitative outcomes [Primary care outcomes for HMR only]
- February 2018 – Interim report. Including:
  - Qualitative analysis
  - Quantitative analysis of data provided in December [Primary and secondary care outcomes in HMR and Oldham]
- September 2018 – Final report. Including:
  - Full quantitative analysis of data up to June 2018
  - Full qualitative analysis
  - Discussion of overall findings from both qualitative and quantitative analysis

Timescales for quantitative reports are determined by data release.

**Interventions (according to the TIDIER guidelines)^[26](#_3as4poj)^**

| *What* | *Risk stratification* |
| --- | --- |
| *Who* |  |
| *How and where* |  |
| *When and how much* |  |
| *Tailoring* |  |
| *Modifications* |  |
| *How well* |  |
| *What* | *Evidence into practice* |
| *Who* |  |
| *How and where* |  |
| *When and how much* |  |
| *Tailoring* |  |
| *Modifications* |  |
| *How well* |  |
| *What* | *Closercare* |
| *Who* |  |
| *How and where* |  |
| *When and how much* |  |
| *Tailoring* |  |
| *Modifications* |  |
| *How well* |  |

**References**

1. Bodenheimer T, Wagner E, Grumbach K. Improving primary care for patients with chronic illness: the Chronic Care Model, part 2. *JAMA* 2002;288:1909-14.

2. Panagioti M, Richardson G, Small N, Murray E, Rogers A, Kennedy A, et al. Self-management support interventions to reduce health care utilisation without compromising outcomes: a systematic review and meta-analysis. *BMC Health Services Research* 2014;14:356.

3. Wagner E. Chronic disease management: What will it take to improve care for chronic illness? *Effective Clinical Practice* 1998;1:2-4.

4. Wagner E, Grothaus L, Sandhu N, Galvin M, McGregor M, Artz K, et al. Chronic care clinics for diabetes in primary care: a system-wide randomized trial. *Diabetes Care* 2001;24(4):695-700.

5. Stokes J, Panagioti M, Alam R, Checkland K, Cheraghi-Sohi S, Bower P. Effectiveness of case management for 'at risk' patients in primary care: a systematic review and meta-analysis. *PLoS One* 2015;10(7):e0132340.

6. Gravelle H, Dusheiko M, Sheaff R, Sargent P, Boaden R, Pickard S, et al. Impact of case management (Evercare) on frail elderly patients: controlled before and after analysis of quantitative outcome data. *BMJ* 2007;334:31.

7. Cartwright M, Hirani S, Rixon L, Beynon M, Doll H, Bower P, et al. Effect of telehealth on quality of life and psychological outcomes over 12 months (Whole Systems Demonstrator telehealth questionnaire study): nested study of patient reported outcomes in a pragmatic, cluster randomised controlled trial. *BMJ* 2013;346:f653.

8. Henderson C, Knapp M, Fernández J, Beecham J, Hirani S, Cartwright M, et al. Cost effectiveness of telehealth for patients with long term conditions (Whole Systems Demonstrator telehealth questionnaire study): nested economic evaluation in a pragmatic, cluster randomised controlled trial. *BMJ* 2013;346:f1035.

9. Henderson C, Knapp M, Fernández J-L, Beecham J, Hirani SP, Beynon M, et al. Cost-effectiveness of telecare for people with social care needs: the Whole Systems Demonstrator cluster randomised trial. *Age and Ageing* 2014.

10. Best A, Greenhalgh T, Lewis S, Saul J, Carroll S, Bitz J. Large scale transformation in health care: a realist review. *Milbank Quarterly* 2012;90(3):421-56.

11. Hibbard J, Gilburt H. Supporting people to manage their health: An introduction to patient activation. London, 2014.

12. Kivelä K, Elo S, Kyngäs H, Kääriäinen M. The effects of health coaching on adult patients with chronic diseases: A systematic review. *Patient Education and Counseling* 2014;97(2):147-57.

13. Dennis S, Harris M, Lloyd J, Powell Davies G, Faruqi N, Zwar N. Do people with existing chronic conditions benefit from telephone coaching? A rapid review. *Australian Health Review* 2013;37:381-88.

14. Hutchison A, Breckon J. A review of telephone coaching services for people with long-term conditions. *Journal of Telemedicine and Telecare* 2011;17:451-58.

15. Barrett D. Remote Monitoring in Torbay: User feedback on the Closercare Patient Monitoring Service: University of Hull, 2014.

16. Barrett D. Telehealth in Harrow: Early findings on activity and cost benefits: University of Hull, 2014.

17. Greenwich N. Reducing hospital admission rates for people with diabetes: a systematic approach to improving primary care outcomes. *Quality and Productivity: Proven Case Study*. <http://www.evidence.nhs.uk/qipp:> NHS Greenwich, 2014.

18. Craig P, Dieppe P, Macintyre S, Michie S, Nazareth I, Petticrew M. Developing and evaluating complex interventions: the new Medical Research Council guidance. *BMJ* 2008;337(sep29_1):a1655.

19. Craig P, Dieppe P, Macintyre S, Michie S, Nazareth I, Petticrew M. Developing and evaluating complex interventions: the new Medical Research Council guidance. *BMJ* 2008;337.

20. Raine R, Fitzpatrick R, Barratt H, Bevan G, Black N, Boaden R, et al. Challenges, solutions and future directions in the evaluation of service innovations in health care and public health. *Health Serv Deliv Res* 2016;4(16).

21. Papoutsi C, Boaden R, Foy R, Grimshaw J, Rycroft-Malone J. Challenges for implementation science In: Raine R, Fitzpatrick R, Barratt H, Bevan G, Black N, Boaden R, et al., editors. *Challenges, solutions and future directions in the evaluation of service innovations in health care and public health. Health Services and Delivery Research*, 2016:2016;4(16). pp. 121–32.

22. Kennedy A, Bower P, Reeves D, Blakeman T, Bowen R, Chew-Graham C, et al. Implementation of self management support for long term conditions in routine primary care settings: cluster randomised controlled trial. *BMJ* 2013;346:f2882.

23. Moore GF, Audrey S, Barker M, Bond L, Bonell C, Hardeman W, et al. *Process evaluation of complex interventions: Medical Research Council guidance*, 2015.

24. Geertz C. Thick Description: Toward an Interpretive Theory of Culture. *The Interpretation of Cultures: Selected Essays*. New York: Basic Books, 1973:3-30.

25. O’Neill S, Kreif N, Grieve R, Sutton M, Sekhon JS. Estimating causal effects: considering three alternatives to difference-in-differences estimation. *Health Services & Outcomes Research Methodology* 2016;16:1-21.

26. Hoffmann TC, Glasziou PP, Boutron I, Milne R, Perera R, Moher D, et al. Better reporting of interventions: template for intervention description and replication (TIDieR) checklist and guide. *BMJ* 2014;348.
