# Supplementary material for: The impact of a combinatorial digital and organisational intervention on the management of long-term conditions in UK primary care: a non-randomised evaluation
Source: BMC Health Serv Res. 2019 Mar 12;19:159. doi: 10.1186/s12913-019-3984-6 (PMC6416963; doi:10.1186/s12913-019-3984-6)
Supplement: Supplementary file 2 — Heywood Middleton and Rochdale Long Term Conditions NHS Test-Bed: A service evaluation of implementation and impact. Qualitative study protocol. (DOCX 98 kb) [file 12913_2019_3984_MOESM2_ESM.docx]

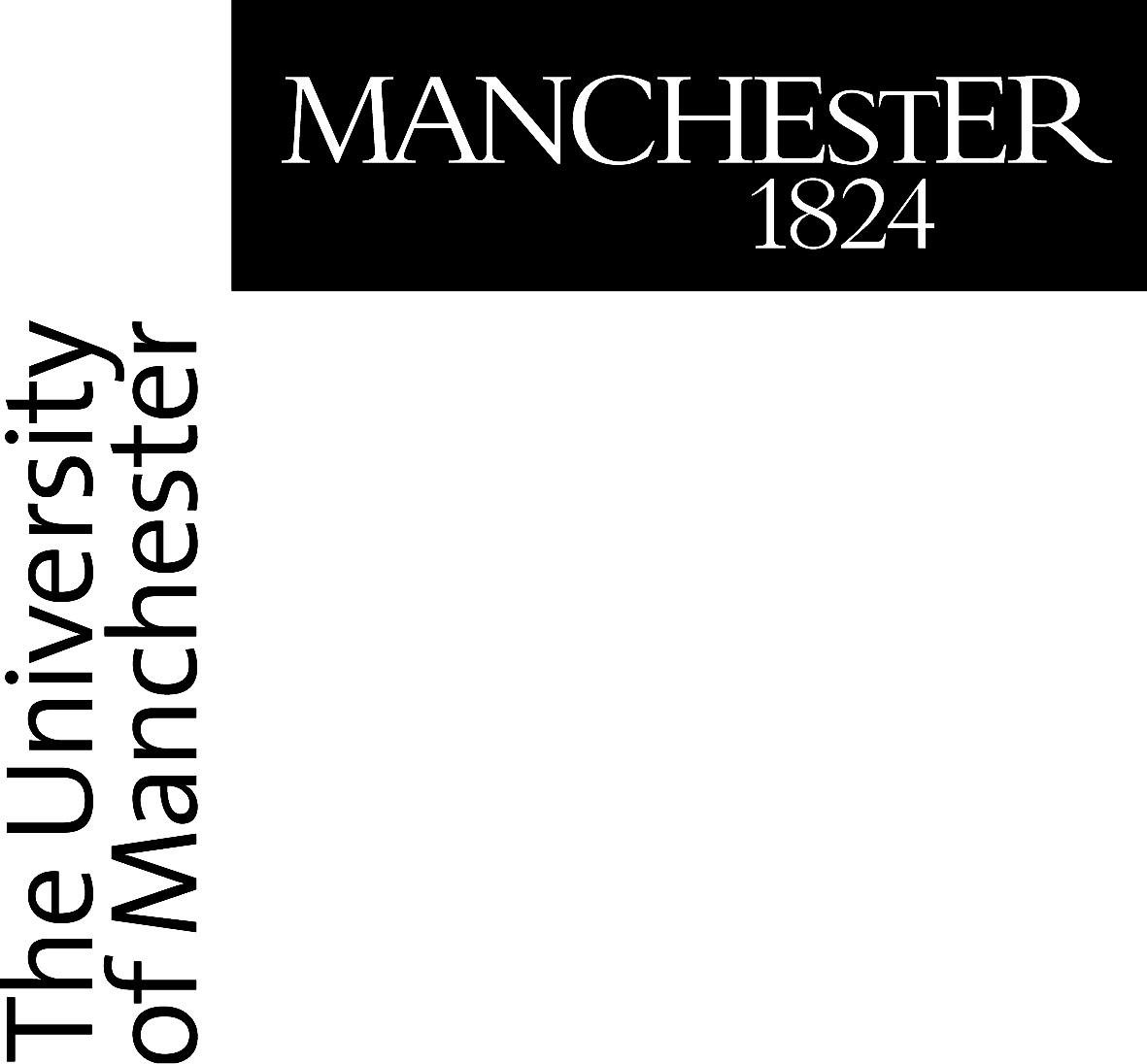


**Heywood Middleton and Rochdale Long Term Conditions NHS Test Bed: A service evaluation of implementation and impact**

Protocol version 1.5 (16/08/2017)

Professor Ruth McDonald

# Part 1

## 1.1 Project summary

NHS Test Beds aim to assess the value of new technologies to healthcare delivery in the UK and have emerged in response to pressures facing the NHS. A test bed involves frontline health and care workers pioneering the use of novel technologies and ways of working to help patients stay well and monitor their conditions themselves at home. One of these Test Beds aims to improve the care of patients with diabetes, heart failure and chronic obstructive pulmonary disease (COPD) by combining clinical audits, population management software, structured training of primary care teams and a patient monitoring and coaching telephone service over a 12-month period in a single Clinical Commissioning Group (CCG). Since similar complex interventions have failed to demonstrate effects because of poor implementation/adherence, this qualitative study will evaluate the implementation of the Test Bed by exploring the extent to which the programme is acceptable to health care professionals and the extent to which professionals’ behaviour/s is/are in line with that intended by the programme leaders.

Observation and longitudinal interviewing of key stakeholders (NHS professionals and other staff) will explore these issues in-depth to identify barriers and facilitators to implementing the Test Bed, and explore if, and how, these factors change during the Test Bed. With participants’ consent, qualitative interviews will be audio-recorded and transcribed by a University-approved supplier. The recording device is not encrypted, but the audio file will be transferred by the researcher to a secure server at the University of Manchester and the original file will be deleted from the audio device. Field notes will be made during observations. NVivo software will support the thematic analysis of interview transcripts, field notes and reflections. Documentary analysis will enrich the thematic analysis to provide an in-depth description of events.

Ethical approval will be obtained through the University of Manchester Research Ethics Committee (UREC) If the decision is taken to roll out the Test Bed, the results of this study will inform its implementation beyond the CCG.

### Study summary at a glance

| Study Title | Heywood Middleton and Rochdale Long Term Conditions NHS Test Bed: A service evaluation of implementation and impact |
| --- | --- |
| Short title | HMR NHS Test Bed - qualitative evaluation (version 1) |
| Study Design | Qualitative process evaluation using observations and interviews |
| Study Participants | Staff at GP practices, the CCG and MSD involved in implementing the Test Bed |
| Planned Size of Sample (if applicable) | N/A |
| Follow up duration (if applicable) | N/A |
| Planned Study Period | 18 months |
| Research Question/Aim(s) | The aim of the study is to evaluate the implementation of the Heywood Middleton and Rochdale Long Term Conditions NHS Test Bed package. The study will achieve this by addressing the following objectives:   - To what extent is the Test Bed programme acceptable to health care professionals? - To what extent is/are the behaviour/s of health care professionals in line with that intended by the programme leaders? |

## 1.2 General information

*Protocol title*: Heywood Middleton and Rochdale Long Term Conditions NHS Test Bed: A service evaluation of implementation and impact

*Protocol version*: 1.1

*Protocol date*: 02/06/2017

*Sponsor*: April Lockyer, The University of Manchester, Oxford Road, Manchester, M13 9PL. Telephone: 0161 275 5436

*Chief/Principal Investigator*: Professor Ruth McDonald (Manchester Business School, Centre for Primary Care, University of Manchester). Email: Ruth.McDonald@mbs.ac.uk

*Co-investigator*: Sarah Darley (Centre for Primary Care, University of Manchester). Email: Sarah.Darley@manchester.ac.uk

*Other institutions*: NHS Heywood, Middleton and Rochdale Clinical Commissioning Group (HMR CCG), 3rd Floor Number One Riverside, Smith Street, Rochdale, OL16 1XU. Telephone: 01706 652 853.

*Funder*: NHS England via HMR CCG, Test Beds programme funding stream

## Rationale & background information

The burden of disease is shifting to long-term conditions, and there is significant interest in the development of interventions that can improve care for these conditions [1]. Preferably, these interventions need to meet the ‘triple aim’ of improving outcomes, reducing costs, and enhancing patient experience, although at a minimum there is a need to develop interventions that can reduce cost without compromising outcomes and experience [2].

A variety of interventions have been tested, including:

- Improvements in the quality of care for long-term conditions through adoption of clinical guidelines and other standard quality improvement methods [3, 4].
- Use of risk stratification models, linked to clinical interventions like case management [5, 6].
- Telehealth, using a variety of models including in-home sensors and more active remote monitoring of long-term conditions [7-9].

Evidence of effectiveness is variable for all these approaches, and it is unclear what interventions are effective, the populations they are effective in, or the contexts in which they can best achieve change.

There is also increasing consensus that attempting to significantly reduce care costs requires ‘large scale transformations’ rather than the delivery of discrete interventions [10].

The NHS Five Year Forward View first proposed new NHS Test Beds, which were designed to assess the value of new technologies. A test bed was described as an area where:

‘Frontline health and care workers …. will pioneer and evaluate the use of novel combinations of interconnected devices such as wearable monitors, data analysis and ways of working which will help patients stay well and monitor their conditions themselves at home’

In 2016, the NHS announced the NHS Test Bed Programme (<https://www.england.nhs.uk/ourwork/innovation/test-beds/>). Heywood, Middleton and Rochdale Clinical Commissioning Group (HMR CCG) successfully led a submission to the NHS England Test Bed programme looking to pioneer and evaluate the ‘use of novel combinations’ of interconnected innovations to improve patient outcomes at the same or lower cost, when compared to routine care (<https://www.england.nhs.uk/wp-content/uploads/2015/03/test-bed-prospectus.pdf>). The HMR CCG Test Bed is designed to improve how GP practices manage patients with diabetes, heart failure and COPD, and has three core components:

1. Clinical audit and population management software for primary care healthcare professionals (called ‘MSDi’)
2. A structured 12-month training programme that ‘upskills’ primary care teams (called ‘Evidence into Practice’)
3. Remote telehealth monitoring service and coaching for up to 1500 patients with Heart Failure or COPD (called ‘Closercare’)

The combination of these interventions, delivered over a 12-month period in a single CCG, is expected to deliver greater impact on health care utilisation (especially hospital admissions) than the individual components alone.

The combination of components in the Test Bed has the characteristics of a ‘complex intervention’ (as defined by the MRC) [11], which are by their nature difficult to implement, as they require changes in service delivery and organisation to be undertaken by professionals and patients in a relatively short period of time [11-13]. There are many examples in the literature of similar health care models which have failed to demonstrate effects because of poor implementation or adherence [14]. It is therefore important to qualitatively evaluate the implementation of the Test Bed under investigation to understand how the programme’s outcomes may have been affected by what was implemented. Furthermore, any roll out to other sites will require guidance on how to implement this type of intervention in such a way as to increase chances of success.

## 1.4 Study goals and objectives

The aim of the study is to evaluate the implementation of the Heywood Middleton and Rochdale Long Term Conditions NHS Test Bed package. The study will achieve this by addressing the following objectives:

- To what extent is the Test Bed programme acceptable to health care professionals?
- To what extent is/are the behaviour/s of health care professionals in line with that intended by the programme leaders?

## 1.5 Study Design and Methodology

The qualitative study is a process evaluation using observations and interviews with a range of staff involved in the implementation of the Test Bed within HMR CCG. The research population will consist of members of staff at HMR CCG, MSD and GP practices within the CCG. HMR CCG is one of the most deprived areas in England, with high prevalence of diabetes, heart failure and COPD. According to local data, two per cent of patients account for 17% of the healthcare spend. We will select eight GP practices in which to conduct the qualitative research. This number is a compromise between the breadth required to capture sufficient variation and the depth needed for detailed exploration. The research sites will be purposively sampled based on practice size and ensuring that all change managers are included.

The MRC defines a process evaluation as:

‘A study which aims to understand the functioning of an intervention, by examining implementation, mechanisms of impact, and contextual factors. Process evaluation is complementary to, but not a substitute for, high quality outcomes evaluation’ [15].

Implementation is defined as: ‘the process through which interventions are delivered, and what is delivered in practice. Key dimensions of implementation include: implementation process (the structures, resources and mechanisms through which delivery is achieved); fidelity (the consistency of what is implemented with the planned intervention); adaptations (alterations made to an intervention in order to achieve better contextual fit); dose (how much intervention is delivered); reach (the extent to which a target audience comes into contact with the intervention)’ [15].

To assess implementation, we will use a combination of observation and interviews. We will conduct up to 90 interviews in total. We will interview professionals (n=4-5) at each practice early on in the evaluation and interview these same professionals towards the end of the process (up to 80 interviews in total). In addition, we will interview staff from MSD and HMR CCG (e.g. members of the Change Team who are involved in staff training and supporting practice staff more generally). Qualitative research interviews will be conducted either face to face at participants’ places of work, or by telephone, and will last up to 1 hour; interviews will take place at participants’ convenience. Potential interview participants within GP practices will be selected by convenience, pragmatic sampling. Potential interview participants outside GP practices (staff at CCG and MSD) will be purposively sampled based on their involvement in setting up the Test Bed. Purposive sampling (based on emerging themes) will be used latterly in response to initial recruitment, data collection and data analysis.

We will also observe professionals in each of the study sites and members of staff at HMR CCG and MSD in meetings/workshops/sessions that focus on the implementation of the Test Bed. Observations will be identified based on whether or not meetings/sessions/workshops focus on the Test Bed. Potential participants attending these meetings/sessions/workshops will therefore be purposively sampled. Purposive sampling (based on emerging themes) will be used subsequently in response to initial recruitment, data collection and data analysis.

Data collection and analysis will be undertaken concurrently. In addition to notes and reflections on observations and interview transcripts, we will use documentary analysis to provide ‘thick description’ of events [16]. Analysis will initially involve coding transcripts using NVivo software and identifying themes. We will use the programme’s intended mechanisms of change to help inform our understanding of the data and focus for data collection. We will not use a specific theoretical framework, but will adopt a sufficiently broad approach to ensure that we do not miss important issues or factors by restricting analysis in too narrow a fashion.

Individuals outside the research team will make the first approach to potential participants; the research team will not contact potential participants in the first instance; we will only contact potential participants that have contacted us and provided their contact details. All potential interview participants will be asked to contact the research team if they are interested in taking part in the study)’. Potential participants for observations will be able to object/agree to being observed by contacting the chair, who will inform the research team, or telling the researcher face to face immediately before the proposed observation. Individuals outside the research team will send reminder emails to potential participants. Written consent will be obtained from interview participants and verbal consent will be obtained for observations (see1.15 Ethical and Regulatory Considerations).

Inclusion criteria: Any staff in the selected GP practices or employed by HMR CCG or MSD involved in the implementation/delivery of the Test Bed will be eligible to participate in the qualitative interviews. Similarly, any participants outside and within these practices attending meetings/workshops/sessions that are relevant to the study’s aims and objectives will be eligible for observations.

Exclusion criteria: Staff at practices, CCG and MSD without a strong verbal command of the English language will not be eligible to participate in the study.

Withdrawal criteria: participants will be able to withdraw from the study without giving a reason. Should participants wish, their data can be withdrawn and destroyed prior to publication of results.

Ethical and site approvals will be submitted in May/Jun 2017. The empirical research will commence following approval and finish by 31^st^ October 2018. The study’s schedule is outlined in 1.12 Duration of the Project.

## 1.6 Safety Considerations

For staff observations, the main burden for participants will be the intrusion of having events observed by a researcher. To minimise this, the researcher will be flexible to the times of scheduled events (e.g. practice meetings and training sessions) when conducting observations. Participants will be informed about the research beforehand and able to decline to participate. If participants object to observation, the event will not be observed.

For staff interviews, the main burden for participants will be giving time to be interviewed. Given that busy staff might find it hard to make time for interviews, researchers will be flexible in finding a time and place that is convenient for participants, offer telephone interviews, and remind potential participants that their participation is voluntary.

While the risk is low, we will follow the University’s Lone working policy and risk assessment for off-site work in the UK to ensure our safety. The Lone Working policy includes details of a 'buddy system' (including contingent arrangements for annual leave, bereavement and sickness) and the escalation process. Before travelling to each site, the lone worker will have informed the buddy of the location, date, and time of the interview/observation. Anticipated times of arrival and return will be agreed and the lone worker will contact the buddy at these times. Where the lone worker fails to adhere to agreed arrangements, the buddy will first attempt to contact the lone worker. Where this is unsuccessful, the buddy will escalate to University Security who will advise if police involvement is needed.

## 1.7 Follow-Up

Routine follow-up of participants is not planned for this study because the topic is non-sensitive.

##

## 1.8 Data Management

Data generated by the study will be managed in accordance with the University's Data Protection Policy, Records Management Policy and Information Security Policy.

Manual personal data will be stored in a locked cabinet, in an office that is locked when unattended, at the University of Manchester. Confidential waste will be destroyed in accordance with the relevant University procedures. Access to the information will be limited to the study staff and investigators and relevant regulatory authorities.

Digital data will be encrypted, password-protected and stored on a secure RDS server at the University of Manchester; the login credentials of investigators are required to access the server, thereby restricting access only to those members of the research team that need it (Professor McDonald and Dr Darley). The University of Manchester’s Research Data Management Service (RDMS) provides robust, managed, secure, replicated storage, and allows researchers to store, manage and curate their data. All electronic data will be stored and automatically backed up daily on secure University of Manchester RDS server, allowing data to be recovered in the event of an incident. Files stored on this service can be considered secure. For example, files corrupted or accidentally deleted can be recovered for up to 35 days.

Personal identifiable data such as participants’ names and work details (telephone numbers, email addresses) will be stored as password-protected digital files on the secure RDS server. To enable these data to be stored separately to interview and observation data, each participant in the study will be assigned an identification number; the anonymisation key linking participants to the interview/observation data will be stored in a separate location.

Whenever possible, personal data will be encrypted and password-protected during electronic transfer. In the following situations, it will not be possible to encrypt personal data during electronic transfer data: 1) Digital audio files will be wire-transferred from an audio-recording device to the secure University RDS; 2) Digital audio files will be uploaded to the secure website of the University-approved transcription service from the secure University RDS; 3) Verbatim transcripts will be downloaded from the secure website of the University-approved transcription service to the secure University RDS. The transcription company is based in the UK and managed through a secure website, and has signed a confidentiality agreement with the University to ensure all personal or sensitive data are protected and stored securely during and after transcription has occurred.

Data from interviews, documents and observations will be analysed together using NVivo software. This will take place at the University of Manchester and will be conducted by the research team comprising members of staff at the University of Manchester. All the data will be stored on the University's secure RDS server and team members will have access to it.

Members of the qualitative research team conducting data collection and analysis will therefore have access to personal data during the study. Study data and material may also be looked at by individuals from the University of Manchester, from regulatory authorities or from the NHS Trust, for monitoring and auditing purposes, and this may well include access to personal information. When the primary buddy (Professor McDonald/Dr Darley) is unavailable (sickness, bereavement, annual leave), the ‘contingent’ buddy will need to have access to interview/observation details (participants’ names, interview/observation location, start and end times) in order to perform this role. Professor Bower will perform the ‘contingent’ buddy role in the first instance. If Professor Bower is unavailable, a member of staff at the University of Manchester will perform this role.

The University's Data Protection Policy, Records Management Policy and Information Security Policy will inform arrangements for storing research data after the study has ended. Research data generated by the study will be stored for 10 years. Manual data will be stored physically in locked filing cabinets at the University of Manchester; digital data will be archived using the RDMS. Consent forms will be retained as essential documents for 10 years, but items such as contact details will be deleted as soon as they are no longer needed. No other identifiable manual data will be stored after the study has ended. Digital audio files will be stored for a minimum of 5 years (maximum 10) before being destroyed; anonymised copies of digital transcripts will be stored for 10 years on the RDS server. All other digital data that will be stored/accessible via the RDMS service after the study will be anonymised (e.g. transcripts, field notes). The anonymisation key will be archived and destroyed at the same time as digital audio files.

In order to perform her data custodian role beyond the study, only Professor McDonald will have access to archived digital and manual data. The University of Manchester will cover the costs of archiving. Archiving arrangements will be water-, pest- and fire-proof. Archived data will be destroyed in accordance with the relevant University policies.

Data generated by the study will not be shared for other research and/or teaching purposes.

## 1.9 Quality Assurance

The study will be subject to the audit and monitoring regime of the University of Manchester.

## 1.10 Expected Outcomes of the Study

The results will inform any roll out of the Test Bed beyond HMR CCG; however, the concurrent quantitative analysis will determine the impact of the Test Bed and therefore inform any decision about whether or not to roll it out beyond HMR CCG. The qualitative data will be considered after the results of the quantitative analysis are known in order to aid interpretation of results.

## 1.11 Dissemination of Results and Publication Policy

The results of the study will be published as a full journal paper in an academic peer reviewed journal; a member of the research team will take the lead in publications. All study team members who make a substantive contribution to reading and writing the final report will be granted authorship on the final study report*.* Funders and participants will be acknowledged in publications. Interim and final reports will also be provided to those funding the research and the Test Bed Programme National Evaluation. The final report will be sent to all participants after the study has ended.

The funders will have no role in the collection, management, analysis and interpretation of data; writing of the report; or the decision to submit the report for publication.

## 1.12 Duration of the Project

Ethical and site approvals will be submitted in May/June 2017. The empirical research will commence following approval and finish by 31^st^ October 2018. A detailed monthly timeline is appended.

## 1.13 Problems Anticipated

Recruiting participants, particularly recruiting interview participants in busy GP practices could be problematic because of time restraints on the study population. To minimise these problems, we have developed strong relationships with CCG and MSD staff involved in implementing the Test Bed (e.g. CCG Programme Manager, Change Managers); permission has been granted by the CCG for observations, although consent will also need to be obtained from practices participating in the Test Bed initiative. While Change Managers have explained the planned evaluation, we will engage with practice management staff before any research is conducted at GP practices to develop a working relationship with these gatekeepers. The external legitimisation conferred to the research team by the CCG will help to develop these key relationships at each study site.

## 1.14 Project Management

The Chief/Principal Investigator, Professor Ruth McDonald will make key methodological decisions, recruit and obtain consent from participants, conduct data collection and analysis, and write interim and final report. Professor Ruth McDonald will also provide project oversight and supervise the co-investigator(s) throughout the project and at monthly meetings. The co-investigator(s) will recruit and obtain consent from participants, conduct data collection and analysis, and write interim and final reports. Professor Peter Bower will provide oversight and supervision through monthly meetings.

The sponsor is responsible for securing the arrangements to initiate, manage and finance a study. In practice this means satisfying itself the research protocol, research team and the research environment have passed appropriate scientific quality, satisfying itself that the study has ethical approval before it begins; satisfying itself that arrangements are kept in place for good practice in conducting the study, and for monitoring and reporting, including prompt reporting of suspected unexpected serious adverse events or reactions and ensuring arrangements are in place for insurance and/or indemnity to meet the potential legal liability of the sponsor(s) for harm arising from the research. Decisions relating to data analysis and interpretation, manuscript writing, and dissemination of results will be made by the research team at the University of Manchester. The funder will be kept informed by the team and made aware of results prior to publication but published outputs will contain a disclaimer reflecting the fact that results and related interpretation are those of the researchers and not the funder.

## 1.15 Ethical and Regulatory Considerations

This research will obtain ethical approval from the University of Manchester Research Ethics Committee (UREC) and will not commence until this is given. The research will protect the privacy and personal information of participants. While the methods chosen (interviews, observation and documentary analysis) are unlikely to pose risks to participants, we will take the steps below to minimise risks to participants.

### 1.15.1 Assessment and management of risk

The main issue raised by this study is maintaining participants' confidentiality and protecting the identity of the practices and individuals within the research process. To ensure the confidentiality of personal data, investigators will follow the University's Data Protection Policy, Records Management Policy and Information Security Policy.

While we will anonymise all organisations, the Clinical Commissioning Group (CCG) and MSD will be identifiable because these details and the geographical location of the Test Bed are available in the public domain; however, the confidentiality of participants, GP practices and other organisations will be maintained by anonymising data. Interview transcripts and field notes from observations will be anonymised as soon as possible by replacing the names of individuals with coded identifiers; we will also use generic job titles to describe individuals’ roles and in some cases we will alter certain contextual details in order to preserve anonymity. Organisations (including GP practices) will be anonymised by making reference only to the type of organisation rather than its name. Details of participation will not be given to any person outside of the research team and we will not quote directly from minutes of meetings. This process will be applied to any related documents from meetings and to research outputs to prevent individuals, organisations and practices being identified in reports and publications. Since the anonymisation key will be archived and destroyed at the same time as digital audio files, the data will be fully anonymised when archiving arrangements cease/expire.

To maintain participants’ confidentiality, the identifiable manual and digital data generated by the study will also need to be stored securely. The study’s storage arrangements for ensuring the security of these data are described in

1.8 Data Management.

Written consent will be obtained for interview participants. To obtain informed consent from interview participants, we will ensure that participants understand the purpose and nature of the research, what the research will involve for them, its benefits, and risks and burdens. The investigator will provide clear verbal explanations about the study, discuss the written information sheet with participants and provide satisfactory answers to participants' questions and spend as much time as the person requires. The investigator will check that participants are able to retain the information long enough to make an effective decision and their decision is based on free choice. The importance of voluntary consent that can be withdrawn at any time before publication without giving a reason will be emphasised to participants. In addition, consent will be established immediately before interviews and monitored throughout.

For observations, we will obtain verbal consent from competent adult participants to reduce the burden placed on them. We will contact chairs in advance to request they email those attending the event to obtain consent. This email will explain in concise and clearly understandable terms to all persons invited to take part: who is conducting the research; why it is being conducted (including the true purpose of the research); why they have been asked to take part; what it requires of them (including the amount of time they will be required to commit and what they will have to do); what will happen to the data they provide; whether and how their anonymity and confidentiality will be maintained; and that their participation is voluntary and they are free to withdraw at any time without detriment. We will ensure that oral consent is recorded or witnessed. At the beginning of events, the research team will be introduced by chairs and participants will be able to ask any questions. We will not observe meetings if objections are raised by any of the participants. We will contact chairs ahead of future meetings to repeat the process and give participants, whether or not they have given consent previously, an opportunity to object to the observation. Verbal consent will be confirmed at the start of the every meeting. We will not use posters to inform meeting attendees of observations. Information sheets will be provided before the meeting and will also be available at the time of the observation.

Given that competent adults will be giving consent, coercion is unlikely to present a significant risk in this qualitative study. Individuals outside the research team will make the first approach to potential participants to mitigate the risk of coercion by researchers. Furthermore, the research team will not contact potential participants in the first instance; we will only contact potential participants that have contacted us and provided their contact details. While coercion by senior management can be a risk [17], researchers will be obtaining consent in this study rather than practice management staff, and will ensure participants’ decisions to participate are informed and voluntary. We will minimise the risk of coercion by respecting participants’ autonomy and reminding them that participation is voluntary. For practice-based staff interviews, decisions about participation will be concealed from anyone outside the qualitative research team to minimise the risk of coercion by practice management staff. To achieve this, practice management staff will send reminders to all potential participants.

### 1.15.2 Research Ethics Committee: review & reports

Before the start of the study, approval will be sought from University of Manchester Research Ethics Committee (UREC) for the study protocol, informed consent forms and other relevant documents.

Substantial amendments that require review by UREC will not be implemented until the REC grants a favourable opinion for the study and where necessary reviewed and accepted by R&D departments, and/or other research governance mechanisms.

All correspondence with the REC will be retained.

It is the Chief Investigator’s responsibility to produce the annual reports as required. The Chief Investigator will also notify the REC of the end of the study.

If the study is ended prematurely, the Chief Investigator will notify the REC, including the reasons for the premature termination.

### 1.15.3 Peer review

The protocol has been peer reviewed by Dr Peter Coventry of University of York.

### 1.15.4 Patient & Public Involvement

This study is focused on staff only and we have no specific PPI group established.

### 1.15.5 Regulatory Compliance

Before any site can enrol staff into the study, the Chief Investigator/Principal Investigator or designee will apply for NHS permission from the site management organisation, HEI or NHS Research & Development (R&D).

For any amendment that will potentially affect a site’s NHS permission, the Chief Investigator/ Principal Investigator or designee will confirm with that site’s R&D department that NHS permission is ongoing (note that both substantial amendments, and amendments considered to be non-substantial for the purposes of REC may still need to be notified to NHS R&D).

### 1.15.6 Protocol compliance

Accidental protocol deviations can happen at any time. They must be adequately documented on the relevant forms and reported to the Chief Investigator and Sponsor immediately.

Deviations from the protocol which are found to frequently recur are not acceptable, will require immediate action and could potentially be classified as a serious breach.

### 1.15.7 Data protection and participant confidentiality

All investigators and study site staff will comply with the requirements of the Data Protection Act 1998 with regards to the collection, storage, processing and disclosure of personal information and will uphold the Act’s core principles.

The arrangements for securely storing electronic and manual data, and who has access to these data, are described in

1.8 Data Management.

The Chief Investigator is the data custodian and will maintain all records and documents regarding the conduct of the study. If the responsible investigator is no longer able to maintain the study records, a second person will be nominated to take over this responsibility. Arrangements for preserving data after the study has ended are described in

1.8 Data Management.

### 1.15.8 Amendments

If the sponsor wishes to make a substantial amendment to the REC application or the supporting documents, the sponsor will submit a valid notice of amendment to the REC for consideration. It is the sponsor’s responsibility to decide whether an amendment is substantial or non-substantial for the purposes of submission to the REC.

The research team in consultation with the funder will be responsible for the decision to amend the protocol. The research team in consultation with the sponsor will be responsible for deciding whether an amendment is substantial or non-substantial taking into account relevant guidance. The amendment history will be tracked using version numbers to identify the most recent protocol version.

# Part 2

## 2.1 Budget

Funds for this study have been secured and have been granted by NHS England via Heywood, Middleton and Rochdale Clinical Commissioning Group (HMR CCG).

These funds will cover the researchers’ costs and project costs (e.g. postage, printing, transcription, telephone). Postage and printing costs associated with this study will be reduced, wherever possible, by sending documents electronically. The Sponsor will provide the audio­recording device, and a computer requiring login credentials and connected to a secure server, along with NVivo data management software. Archiving will be covered by the Sponsor.

Staff at HMR CCG and MSD are supportive of the study and have agreed to perform the described recruitment roles without payment. Recruitment performed by staff at GP practices will not be funded. We will obtain support for these activities from practice management staff before conducting interviews and observations at practices.

## 2.2 Other support for the Project

### 2.2.1 Collaboration with other scientists or research institutions

The study team does not plan to collaborate with other research institutions. However, it will provide a summary of interim findings to the team coordinating the evaluation of the Test Beds programme nationally.

### 2.2.2 Links to other projects

This study is linked to a quantitative evaluation of the NHS HMR Test Bed led by Professor Matthew Sutton (Professor of Health Economics, University of Manchester). The data collection process is intended to inform interpretation of the quantitative findings.

## 2.3 Financing and Insurance

The University of Manchester as research Sponsor indemnifies its staff, research participants and research protocols with both public liability insurance and clinical trials insurance. These policies include provision for indemnity in the event of a successful litigious claim for proven non-negligent harm.

# Part 3

## 3.1 Reference List

1. Bodenheimer T, W.E., Grumbach K. , *Improving primary care for patients with chronic illness: the Chronic Care Model, part 2.* JAMA, 2002. **288**: p. 1909-14.

2. Panagioti M, R.G., Small N, Murray E, Rogers A, Kennedy A, et al. , *Self-management support interventions to reduce health care utilisation without compromising outcomes: a systematic review and meta-analysis.* BMC Health Services Research 2014. **14**: p. 356.

3. Wagner, E., *Chronic disease management: What will it take to improve care for chronic illness?* Effective Clinical Practice, 1998. **1**: p. 2-4.

4. Wagner E, G.L., Sandhu N, Galvin M, McGregor M, Artz K, et al, *Chronic care clinics for diabetes in primary care: a system-wide randomized trial.* Diabetes Care, 2001. **24**(4): p. 695-700.

5. Stokes J, P.M., Alam R, Checkland K, Cheraghi-Sohi S, Bower P, *Effectiveness of case management for 'at risk' patients in primary care: a systematic review and meta-analysis.* PLoS One, 2015. **10**(7).

6. Gravelle H, D.M., Sheaff R, Sargent P, Boaden R, Pickard S, et al, *Impact of case management (Evercare) on frail elderly patients: controlled before and after analysis of quantitative outcome data.* BMJ, 2007. **334**: p. 31.

7. Cartwright M, H.S., Rixon L, Beynon M, Doll H, Bower P, et al, *Effect of telehealth on quality of life and psychological outcomes over 12 months (Whole Systems Demonstrator telehealth questionnaire study): nested study of patient reported outcomes in a pragmatic, cluster randomised controlled trial.* BMJ, 2013. **346**: p. f653.

8. Henderson C, K.M., Fernández J, Beecham J, Hirani S, Cartwright M, et al, *Cost effectiveness of telehealth for patients with long term conditions (Whole Systems Demonstrator telehealth questionnaire study): nested economic evaluation in a pragmatic, cluster randomised controlled trial.* BMJ, 2013. **346**: p. f1035.

9. Henderson C, K.M., Fernández J-L, Beecham J, Hirani SP, Beynon M, et al, *Cost-effectiveness of telecare for people with social care needs: the Whole Systems Demonstrator cluster randomised trial.* Age and Ageing, 2014.

10. Best A, G.T., Lewis S, Saul J, Carroll S, Bitz J, *Large scale transformation in health care: a realist review.* Milbank Quarterly, 2012. **90**(3): p. 421-56.

11. Craig P, D.P., Macintyre S, Michie S, Nazareth I, Petticrew M, *Developing and evaluating complex interventions: the new Medical Research Council guidance.* BMJ, 2008. **337**(sep29_1): p. a1655.

12. Raine R, F.R., Barratt H, Bevan G, Black N, Boaden R, et al, *Challenges, solutions and future directions in the evaluation of service innovations in health care and public health.* Health Serv Deliv Res, 2016. **4**(16).

13. Papoutsi C, B.R., Foy R, Grimshaw J, Rycroft-Malone J. Challenges for implementation science In: Raine R, Fitzpatrick R, Barratt H, Bevan G, Black N, Boaden R, et al., editors, *Challenges, solutions and future directions in the evaluation of service innovations in health care and public health.* Health Services and Delivery Research, 2016. **4** (16): p. 121–32.

14. Kennedy A, B.P., Reeves D, Blakeman T, Bowen R, Chew-Graham C, et al, *Implementation of self management support for long term conditions in routine primary care settings: cluster randomised controlled trial.* BMJ, 2013. **346**: p. f2882.

15. Moore GF, A.S., Barker M, Bond L, Bonell C, Hardeman W, et al, *Process evaluation of complex interventions: Medical Research Council guidance*. 2015.

16. Geertz, C., *Thick Description: Toward an Interpretive Theory of Culture. The Interpretation of Cultures: Selected Essays*. 1973, New York: Basic Books.

17. Mulhall, A., *In the field: notes on observation in qualitative research.* Journal of Advanced Nursing, 2003. **41**(3): p. 306–313.

## Study Flow Chart

| **Stage** | **May-17** | **Jun-17** | **Jul-17** | **Aug-17** | **Sep-17** | **Oct-17** | **Nov-17** | **Dec-17** | **Jan-18** | **Feb-18** | **Mar-18** | **Apr-18** | **May-18** | **Jun-18** | **Jul-18** | **Aug-18** | **Sep-18** | **Oct-18** |
| --- | --- | --- | --- | --- | --- | --- | --- | --- | --- | --- | --- | --- | --- | --- | --- | --- | --- | --- |
| UREC application |  |  |  |  |  |  |  |  |  |  |  |  |  |  |  |  |  |  |
| Sponsor Review |  |  |  |  |  |  |  |  |  |  |  |  |  |  |  |  |  |  |
| UREC approval |  |  |  |  |  |  |  |  |  |  |  |  |  |  |  |  |  |  |
| Sampling and recruitment |  |  |  |  |  |  |  |  |  |  |  |  |  |  |  |  |  |  |
| Observations |  |  |  |  |  |  |  |  |  |  |  |  |  |  |  |  |  |  |
| Initial interviews |  |  |  |  |  |  |  |  |  |  |  |  |  |  |  |  |  |  |
| Data analysis 1 |  |  |  |  |  |  |  |  |  |  |  |  |  |  |  |  |  |  |
| Interim report |  |  |  |  |  |  |  |  |  |  |  |  |  |  |  |  |  |  |
| Sampling and recruitment |  |  |  |  |  |  |  |  |  |  |  |  |  |  |  |  |  |  |
| Follow-up interviews |  |  |  |  |  |  |  |  |  |  |  |  |  |  |  |  |  |  |
| Data analysis 2 |  |  |  |  |  |  |  |  |  |  |  |  |  |  |  |  |  |  |
| Final report |  |  |  |  |  |  |  |  |  |  |  |  |  |  |  |  |  |  |
| Publication |  |  |  |  |  |  |  |  |  |  |  |  |  |  |  |  |  |  |
