# Supplementary figures and images for: The impact of a combinatorial digital and organisational intervention on the management of long-term conditions in UK primary care: a non-randomised evaluation
Source: BMC Health Serv Res. 2019 Mar 12;19:159. doi: 10.1186/s12913-019-3984-6 (PMC6416963; doi:10.1186/s12913-019-3984-6)

**Additional File 4. Graphical analysis of parallel trends for secondary care outcomes**


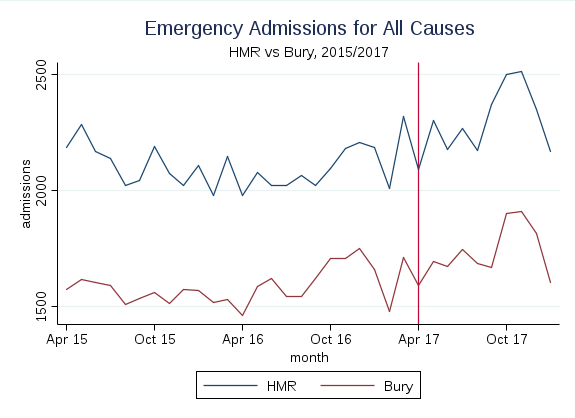

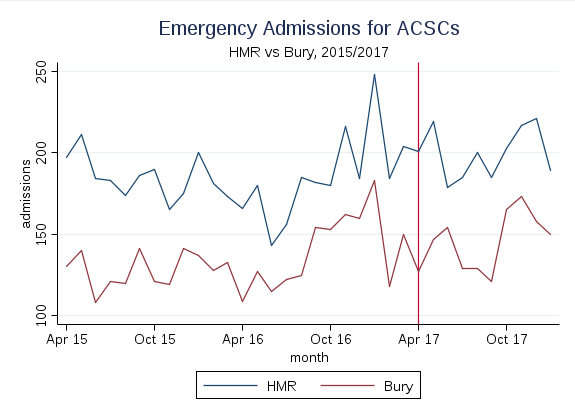

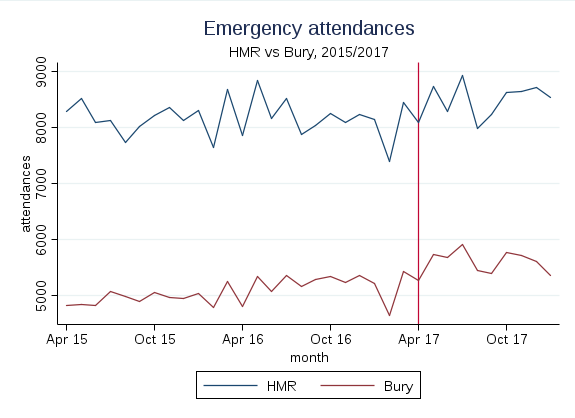

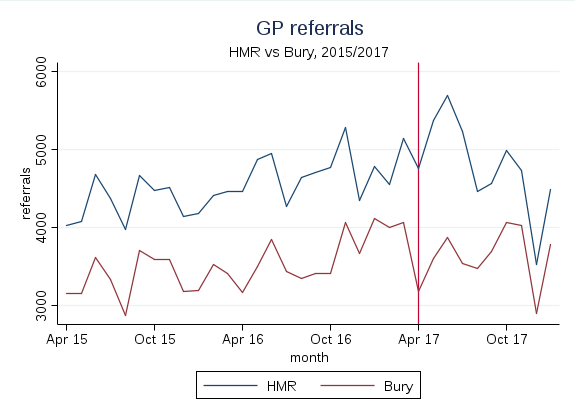

Supplement: Supplementary file 4 — Graphical analysis of parallel trends for secondary care outcomes. Four graphs comparing the trends followed by four secondary care outcomes in both HMR and Bury CCGs. (DOCX 391 kb) [file 12913_2019_3984_MOESM4_ESM.docx]

**Additional File 5. Graphical analysis of parallel trends for primary care outcomes**


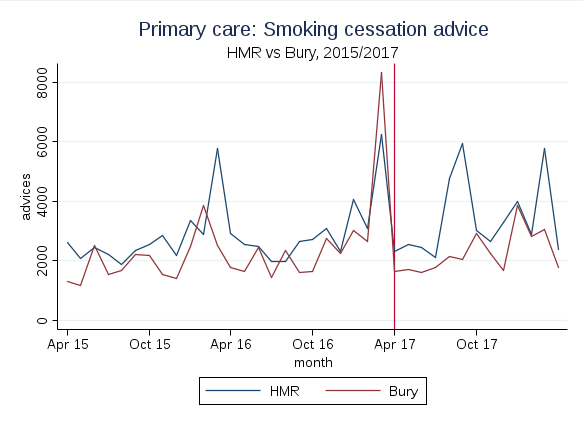

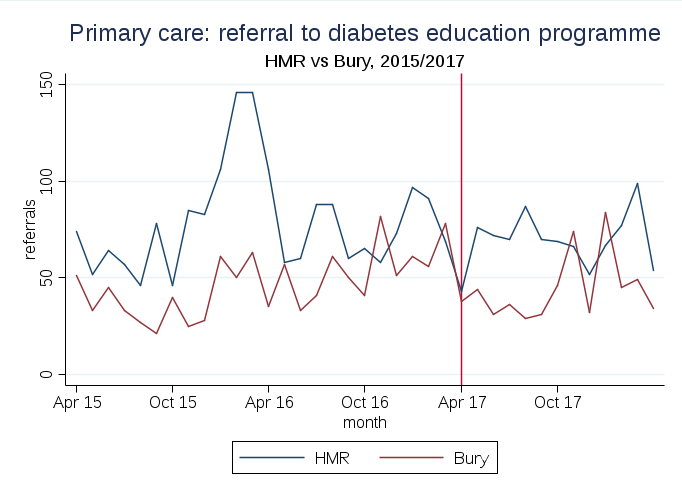

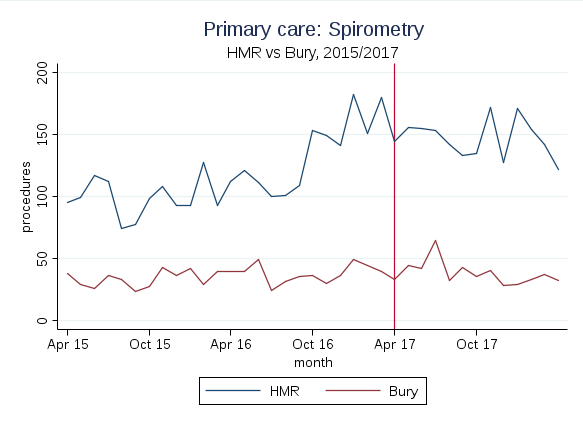

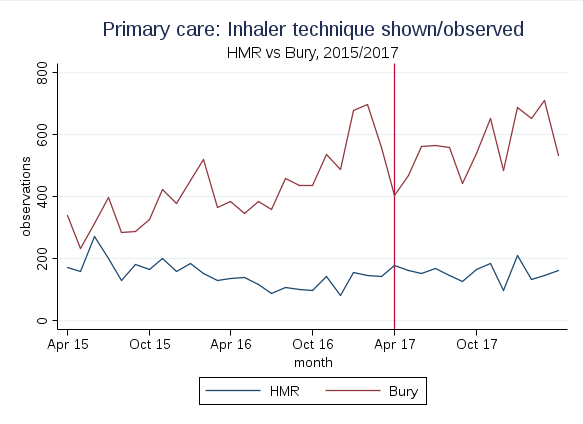

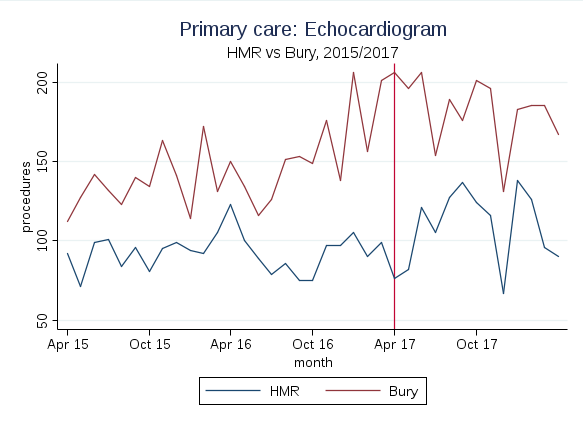

Supplement: Supplementary file 5 — Graphical analysis of parallel trends for primary care outcomes. Five graphs comparing the trends followed by five primary care outcomes in both HMR and Bury CCGs. (DOCX 566 kb) [file 12913_2019_3984_MOESM5_ESM.docx]
